# Reliability and Validity of the Early Years Physical Activity Questionnaire (EY-PAQ)

**DOI:** 10.3390/sports4020030

**Published:** 2016-05-26

**Authors:** Daniel D. Bingham, Paul J. Collings, Stacy A. Clemes, Silvia Costa, Gillian Santorelli, Paula Griffiths, Sally E. Barber

**Affiliations:** 1 Bradford Institute for Health Research, Bradford BD9 6RJ, UK; Paul.Collings@bthft.nhs.uk (P.J.C.); Gillian.Santorelli@bthft.nhs.uk (G.S.); sally.barber@bthft.nhs.uk (S.E.B.); 2 School of Sport, Exercise and Health Sciences, Loughborough University, Leicestershire LE11 3TU, UK; S.A.Clemes@lboro.ac.uk (S.A.C.); P.Griffiths@lboro.ac.uk (P.G.); 3 Centre for Diet and Activity Research (CEDAR), MRC Epidemiology Unit, University of Cambridge, School of Clinical Medicine, Cambridge CB2 0QQ, UK; sc808@medschl.cam.ac.uk

**Keywords:** physical activity, sedentary behaviour, measurement, self-report, preschool, children, accelerometry, ethnicity

## Abstract

Measuring physical activity (PA) and sedentary time (ST) in young children (<5 years) is complex. Objective measures have high validity but require specialist expertise, are expensive, and can be burdensome for participants. A proxy-report instrument for young children that accurately measures PA and ST is needed. The aim of this study was to assess the reliability and validity of the Early Years Physical Activity Questionnaire (EY-PAQ). In a setting where English and Urdu are the predominant languages spoken by parents of young children, a sample of 196 parents and their young children (mean age 3.2 ± 0.8 years) from Bradford, UK took part in the study. A total of 156 (79.6%) questionnaires were completed in English and 40 (20.4%) were completed in transliterated Urdu. A total of 109 parents took part in the reliability aspect of the study, which involved completion of the EY-PAQ on two occasions (7.2 days apart; standard deviation (SD) = 1.1). All 196 participants took part in the validity aspect which involved comparison of EY-PAQ scores against accelerometry. Validty anaylsis used all data and data falling with specific MVPA and ST boundaries. Reliability was assessed using intra-class correlations (ICC) and validity by Bland–Altman plots and rank correlation coefficients. The test re-test reliability of the EY-PAQ was moderate for ST (ICC = 0.47) and fair for moderate-to-vigorous physical activity (MVPA)(ICC = 0.35). The EY-PAQ had poor agreement with accelerometer-determined ST (mean difference = −87.5 min·day^−1^) and good agreement for MVPA (mean difference = 7.1 min·day^−1^) limits of agreement were wide for all variables. The rank correlation coefficient was non-significant for ST (rho = 0.19) and significant for MVPA (rho = 0.30). The EY-PAQ has comparable validity and reliability to other PA self-report tools and is a promising population-based measure of young children’s habitual MVPA but not ST. In situations when objective methods are not possible for measurement of young children’s MVPA, the EY-PAQ may be a suitable alternative but only if boundaries are applied.

## 1. Introduction

The early years (ages 0–5) are vital for establishing healthy lifestyle behaviours including adequate levels of physical activity (PA) and low levels of sedentary time (ST), both of which can have immediate and long-term health impacts [1,2,3]. In the short term, total PA and moderate to vigorous physical activity (MVPA) both seem to be positively, and ST negatively, associated with multiple health outcomes in children (bone health, motor development, cardiovascular risk factors, cognitive development, psycho-social health and healthy adiposity) [3,4,5]. In the long term, low PA and high ST are key risk factors for the onset of non-communicable diseases later in life (*i.e.*, cardiovascular disease and type II diabetes) [6,7,8,9,10]. These long-term influences may partly be explained by levels of PA and ST tracking over time [11].

Objective monitoring tools, such as accelerometers, are the first choice for field-based measures of PA and ST in young children [12]. Accelerometers can reliably and accurately measure the frequency, intensity and duration of young children’s body movement within everyday settings [13,14]. Hnatiuk and colleagues [15] reviewed the results of studies measuring PA (*n* = 40) and ST (*n* = 31) in young children using accelerometers, and found that the daily proportion of time spent sedentary ranged from 34% to 94%, whereas MVPA ranged from 2% to 41% of awake time. Although accelerometers are becoming common-place in published research [14], like all methods, accelerometers have limitations, including expense, participant burden, and the level of expertise required to process and analyse data [12,16,17,18]. These factors may preclude large-scale epidemiological studies of young children’s (in)activity behaviours, which are particularly required within multi-ethnic and economically diverse populations, as there is a need to better understand health inequalities [19,20,21,22].

In 2010, a sub-smaple of the Born in Bradford birth cohort study (BiB-1000) began investigating obesity risk factors for young children living in a multi-ethnic and economically diverse city [23,24]. At the time, there were no available methods deemed appropriate to measure MVPA and ST in this population [24], and an objective measure (such as an accelerometer) was not feasible. For these reasons, a new questionnaire was designed and implemented. A strength of questionnaires over objective measures is the extra contextual information provided about PA and ST (*i.e.*, the activity location and/or type). The new questionnaire generated was the Early Years Physical Activity Questionnaire (EY-PAQ).

The aims of this study were to assess the EY-PAQ’s test re-test reliability, and to determine its validity by comparing EY-PAQ data to accelerometry in a sample of young children from a deprived and multi-ethnic population, where English and Urdu are the predominant languages spoken.

## 2. Materials and Methods

### 2.1. Participants and Setting

The study sample consisted of young children aged 18 months to 4 years and their parents who resided in the City of Bradford, UK. Parents were already recruited as part of a pilot cluster randomized controlled trial [25]. Bradford has an approximate population of 500,000 and is the sixth largest metropolitan area in England [26]. The city is also one of the most ethnically diverse and deprived areas in the UK [26]. Ethical approval for the study was granted by the Bradford Teaching Hospitals Foundation Trust ethics committee, and informed consent was obtained from parents.

### 2.2. Procedure

Parents and children attended two appointments with a trained researcher. The first appointment included completion of the EY-PAQ, measurement of children’s height and weight, and positioning of the Actigraph GT3X+ tri-axial accelerometer (ActiGraph, Pensacola, FL, USA). Parents were instructed on how to fit the accelerometer to their child, which was attached around the waist over the right hip. They were also asked that their child wore the accelerometer during all waking hours for seven consecutive days. The second appointment took place approximately seven days later. Accelerometers were collected and the same researcher-parent pair completed the EY-PAQ.

### 2.3. Measures

#### 2.3.1. The Early Years Physical Activity Questionnaire (EY-PAQ)

The EY-PAQ is a proxy-reported questionnaire that attempts to quantify levels of habitual MVPA and ST in young children. The questionnaire is available in both English and transliterated Urdu (see supplementary material) [26]. Parents were asked to report the frequency and duration of different MVPA and ST activities in which their child engaged during a typical week in the previous month. The activities for MVPA were: (1) playing actively in the house; (2) playing actively in the garden; (3) walking from place to place; (4) engaging in active play causing sweating and increased breathing; (5) playing in the park or playground, and (6) playing at indoor play facilities. The sedentary activities were: (1) colouring, drawing and craft; (2) sitting playing with toys; (3) watching TV/DVDS; (4) playing a non-active computer game; (5) sitting listening or singing to music; (6) reading or being read to; (7) travelling in a buggy/pushchair; (8) being carried while travelling; (9) travelling in the car; and (10) using public transport. A three-stage process was used to calculate daily minutes of MVPA and ST. First, the duration (reporting options were: 1) up to 15 min/day; 2) 16–30 min/day; 3) 31–60 min/day; or 4) free-text for >60 min/day) of each activity was multiplied by the frequency that activity occurred. A pragmatic approach was used with regards to the duration component, as parents tend to over-report PA and under-report ST [27], unless free-text responses exceeding 60 min/day were reported, for the calculation of MVPA, minimum reported durations were used (*i.e.*, 1 min, 16 min, or 31 min), whereas, for ST, the higher values were used (*i.e.*, 15 min, 30 min, and 60 min). Second, the calculated duration of each activity was summed and divided by seven in order to estimate daily minutes of MVPA and ST. Third, daily minutes of MVPA and ST were converted to the proportions of waking time spent in these behaviours, by dividing summed minutes in MVPA and ST, respectively, by 840 min (×100). Fourteen hours (840 min) is typical of a waking day in preschool aged children [28], and is in line with sleep diary data that we have collected from similarly aged children from the source population (data not shown). Proportional values were used as the main outcomes because parents completing the EY-PAQ considered the whole day in which an activity may have taken place, whereas accelerometer measured MVPA and ST was only captured during the time the monitor was worn; this rarely reflected entire waking time. Proportions were therefore used to account for disparities in the reference period [29].

Proxy-reported questionnaires often find large variances in PA and ST due to reporting errors [30]. To non-arbitrarily deal with assumed errors, the validity of the questionnaire was examined using two approaches, firstly by using all EY-PAQ data and secondly only data falling within specific MVPA and ST boundaries. These boundaries reflected the range of published ST and MVPA estimates in young children from objective measures [15]. The boundaries for MVPA were 2% to 41% and for ST 30% to 94% of awake time. The lower boundary for ST was reduced from published value of 34% to account for non-discretionary sedentary behaviours, such as bathroom or meal times, which accelerometry may have captured but the EY-PAQ did not pose questions about.

#### 2.3.2. Accelerometry

The Actigraph GT3X+ (Actigraph Pensacola, FL, USA) is an accurate objective measure that has widely been used to measure young children’s PA and ST [17,31,32,33]. It has been reported to be the favoured device to objectively measure PA in young Bradford children and their mothers [34]. For this study, the accelerometer was set to record data at a sampling rate of 60 Hertz. Raw accelerometer data were downloaded and then transformed into both 5 s and 15 s epoch files. Costa [35] found in a British sample of young children (age 2.9 years (SD 0.60)) that both 10 s and 15 s epochs significantly underestimated ST. From these findings, a new set of accelerometer cut-points were calibrated and validated using 5 s epochs [31]. The Costa cut-points were found to be accurate (criterion measure: direct observation [36]) in estimating young childrens’ ST but were inaccurate in measuring MVPA. Therefore, we incorporated an alternative cut-point to assess MVPA in this study. The Pate MVPA cut-points were chosen because they have been reported to be the most accurate and appropriate to estimate MVPA in young children [37,38]. As ST and MVPA were treated as independent behaviours, the choice of epoch length was set in line with with the procedures of the original calibration studies that developed the ST (Costa: ≤5 counts per 5 s) and MVPA (Pate: 420 counts per 15 s) cut-points [13,31], hence processing data with two epoch lengths. The minimum wear-time for inclusion in the analysis was at least 6 h on any three days, which has been shown to provide reliable activity estimates (ICC = 0.7) in the same population of children used in this study [39]. Non-wear time was defined as ≥10 min of consecutive zero counts [17]. To calculate the proportion of awake time that each child engaged in behaviours, minutes of accelerometer MVPA and ST were divided by wear time, and multiplied by 100.

### 2.4. Data Analysis

Descriptive characteristics are presented for all participants and separately for the validity and reliability study participants.

Test/re-test reliability of the EY-PAQ for both MVPA and ST was assessed using a two-way random model intra-class correlation coefficient (ICC(2,1)) with 95% confidence intervals [40]. For the purposes of this study, coefficient values of 0.01 indicated “poor” agreement, 0.01 to 0.20 “slight” agreement, 0.21 to 0.40 “fair” agreement, 0.41 to 0.60 “moderate” agreement, 0.61 to 0.80 “substantial” agreement and 0.81 to 1.00 “almost prefect” agreement [41,42].

For the validity analysis, assessment of whether sex, ethnicity and language modified the relationships between the EY-PAQ and accelerometer MVPA and ST estimates was performed using multiple linear regression analyses. If any demographic variable was found to modify relationships, subsequent analyses were stratified by the influencing variable. All tests were conducted using proportions data, with and without boundaries. Spearman rank correlations (rho) were applied to assess the correlations between the EY-PAQ and accelerometer data. Bland–Altman plots [43] were assembled to assess the agreement between EY-PAQ and accelerometry (before and after applying boundaries). Differences (error) between EY-PAQ and accelerometer estimates of MVPA and ST were calculated (error = EY-PAQ − Actigraph) and plotted against the mean MVPA and ST values of accelerometry and EY-PAQ. The mean difference and direction of systematic error were examined using Pearson error correlations (error = *x*-axis, Actigraph = *y*-axis). Heteroscedasticity was examined using Breusch–Pagan/Cook–Weisberg Tests; where heteroscedasticity was present, heteroscedastic ratio limits of agreement (LOA) were calculated on the log scale [44,45].

All tests (reliability and validity) were performed using proportions (*i.e.*, the % of awake time in MVPA or ST), but to ease interpretation of the data and enable comparisons with other published questionnaires, proportions were converted back into minutes based upon a waking day of 840 min [28]. Analyses were conducted using SPSS for windows (version 22) and STATA (version 13), and alpha significance was defined as *p* ≤ 0.05.

## 3. Results

**Figure 1:**
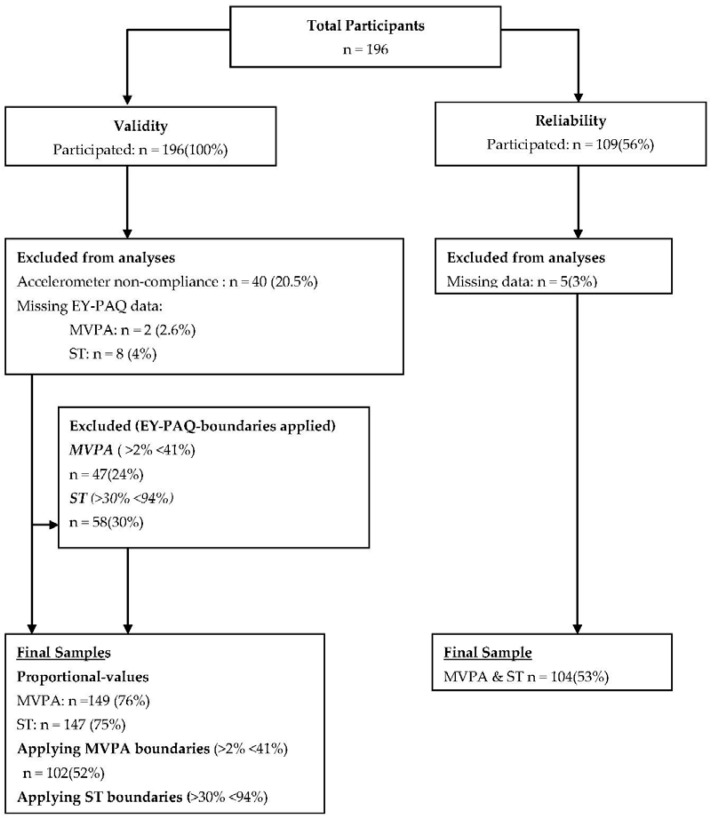
Flow diagram outlining the number of included and excluded participants for the validity and reliability analyses.

The demographic characteristics of children are reported in Table 1. In total, 196 children and their parents took part in the validity analysis and 109 took part in the reliability analysis. A breakdown of the number of participants in each of the components of the study, including details of exclusions, is outlined in Figure 1. The mean age was 3.2 years (SD ± 0.8), 50.5% were boys and 49% South Asian (Table 1). Most questionnaires were conducted in English (79.6%) and with mothers (98%). There were no significant differences in the sex, ethnicity or age of participants included and those excluded from any aspect of the study. Linear regression models found no significant interactions by sex, ethnicity or language, for the relationships between EY-PAQ and accelerometry; therefore, analyses were not stratified.

### 3.1. Reliability

The average number of days between the completion of the questionnaire at Test 1 and Test 2 was 7.4 days, ranging from five to 12 days.

Table 2 shows the results of the test/re-test reliability analyses. There was fair agreement for test/re-test reliability of MVPA measured by the EY-PAQ (ICC(2,1) = 0.35, 95% CI: 0.17–0.50)). For ST, reliability was moderate (ICC(2,1) = 0.47, 95% CI: 0.30–0.61)).

### 3.2. Validity

**Figure 2:**
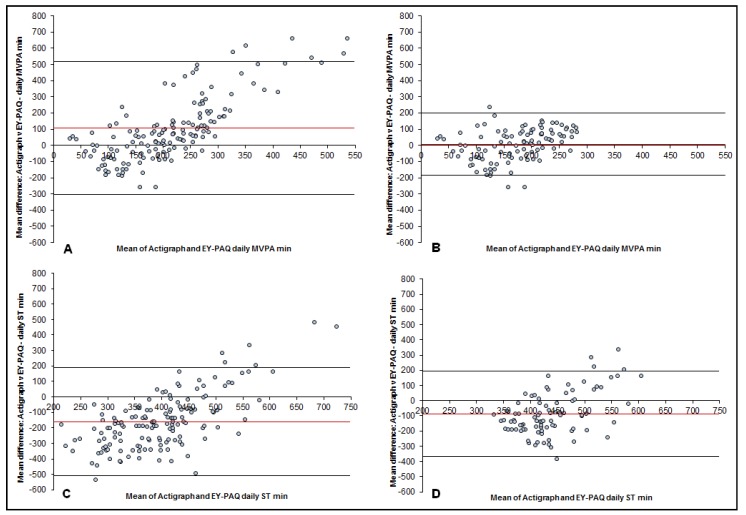
Bland–Altman plots for the difference between time spent in moderate to vigorous physical activity (MVPA) and sedentary time (ST) measured by accelerometry and the Early Years Physical activity questionnaire (EY-PAQ); plotted against the mean average time spent in MVPA and ST measured by the Actigraph and EY-PAQ. (**A**): MVPA plot for EY-PAQ-MVPA no boundaries applied. Mean difference: 106.3 mins/day; 95% limits of agreement (LOA): −303.7, +516.4. (**B**): MVPA plot with EY-PAQ-MVPA boundaries applied. Mean difference: 7.1 mins/day; LOA: −185.9, +200.1. (**C**): ST plot for EY-PAQ-ST no boundaries applied. Mean difference: −160.0 mins/day; LOA: −509.9, +190.0. (**D**): ST plot with EY-PAQ-ST boundaries applied. Mean difference: −87.5 mins/day; LOA: −376.6, +192.7.

Agreement between MVPA and ST estimated by the EY-PAQ and accelerometry are shown in Figure 2. For MVPA, the mean difference was 7.1 min/day (LOA: −185.9 ± 200.1), and, for ST, the mean difference was −87.5 min/day (LOA: −376.6 ± 192.7).

The only significant correlation between the EY-PAQ and accelerometer was the proportion of time spent in MVPA after applying boundaries (Table 3). Error correlations for all values were found to be statistically significant (Table 3). The significant correlations highlight that systematic error existed. Breusch–Pagan/Cook–Weisburg tests found heteroscedasticity to be present (*p* < 0.05) in the MVPA values and in the ST values. The MVPA mean bias on the ratio scale found MVPA was overestimated by 20% (1.20) and when the boundary-value was applied this was reduced to an overestimation of 3% (1.03). The ratio mean bias for ST found that it was overestimated by 72% (1.72) which was reduced to an overestimation of 26% (1.26) when the EY-PAQ boundaries were applied. The ratio-limits of agreement were wide for all results (Table 3).

## 4. Discussion

This study examined the reliability and validity of a new activity questionnaire (EY-PAQ) in a sample of young children from a diverse ethnic background where parents spoke either English or Urdu. Findings of the current study show the EY-PAQ has fair reliability for MVPA and moderate reliability for ST. The validity of the EY-PAQ was assessed against accelerometry. A small mean difference and significant correlation was found for MVPA after applying boundaries, leading to the EY-PAQ being an acceptable population method to measure young children’s habitual MVPA. For ST, the mean difference was large and the correlation coefficient non-significant. This was true even after applying boundaries to the data, it therefore appears that the EY-PAQ is not a suitable population measure of ST.

The EY-PAQ is a new tool which measures the habitual levels of young children’s MVPA and ST. Other similar tools which have been compared to accelerometry are the Preschool-Age Physical Activity Questionnaire (Pre-PAQ) which was developed in Sydney, Australia and measures MVPA, light activity and ST [27], and the Children’s Physical Activity Questionnaire (C-PAQ) which was developed in Cambridge, UK and measures MVPA and total PA [44]. The test/re-test reliability of the MVPA component of the EY-PAQ was found to be fair and acceptable [41,42]. Sedentary time had a greater ICC value than MVPA (0.47 and 0.35). In comparison to other published questionnaires, the EY-PAQ’s ST ICC was similar to that of the Pre-PAQ’s [27] (0.44). The EY-PAQ’s MVPA reliability coefficient was lower than the Pre-PAQ’s (0.54) and C-PAQ’s [44] (0.39). It is perhaps unsurprising that reliability of all questionnaires were low compared to the ‘almost perfect’ criteria of ICC = 0.8. Children’s PA tends to be highly variable [12], which means levels of MVPA and ST could be very different from one week to the next, thus affecting test/re-test results.

With regards to validity, like the other questionnaires, differences in MVPA and ST were seen between the EY-PAQ and accelerometer. For MVPA, initial results of the EY-PAQ validity assessment revealed larger error values in comparison to the Pre-PAQ and C-PAQ. The EY-PAQ overestimated MVPA by 106.3 min/day, compared to the Pre-PAQ which overestimated MVPA by 50.1 min/day, while the CPAQ underestimated MVPA by −76.5 min. With regards to ST, the EY-PAQ underestimated daily sitting by 160 min/day, which was a smaller difference to the Pre-PAQ’s mean difference of −208.6 min/day. However, when the EY-PAQ boundaries were applied differences with accelerometry were reduced to 7.1 min for MVPA and −87.5 min for ST. Like the present study, Corder, van Sluijs [42] also assessed the heteroscedasticity of the C-PAQ and reported the anti-logged ratio limits of agreement. Results showed the C-PAQ at best (depending upon accelerometer cut-points) underestimated MVPA by 32%, which is a greater level of error than the 3% overestimation witnessed with the EY-PAQ after applying boundaries to the data. Limits of agreement for the EY-PAQ, like the Pre-PAQ and CPAQ, were wide but improved with application of boundaries. Applying the EY-PAQ boundaries also improved the correlation coefficient between the two measures for both MVPA and ST. Before the application of boundaries the EY-PAQ had low, non-significant coefficients (MVPA (rho = 0.03, *p* ≥ 0.05) and ST [rho = 0.02, *p* ≥ 0.05]) when compared to the accelerometer. After applying boundaries, the EY-PAQ’s validity coefficients increased (MVPA: rho = 0.30; ST = 0.19). The MVPA coefficient was statistically significant, thus the EY-PAQ successfully ranked young children’s MVPA after applying boundaries. The CPAQ also found that it could rank young children’s MVPA (rho = 0.42, *p* ≤ 0.05). The EY-PAQ’s ST coefficient after applying boundaries increased to 0.19, but was still statistically non-significant.

There are numerous reasons that could explain the differences in the reliability and validity coefficients seen between the EY-PAQ, CPAQ and Pre-PAQ. One reason is that the instruments vary in design and question structure. Another possible reason could be the differences in socio-demographic characteristics of the samples used in the three studies. The Pre-PAQ sample consisted of mainly high socio-economic status, white, English-speaking parents and children from Australia. The samples from both the EY-PAQ and C-PAQ validity studies were located in the UK, but the children in the current study were from communities of high deprivation [23], with a high ethnic mix and two different primary languages. Little is known about the sample of the C-PAQ study, other than the sample was from an affluent part of the UK (Cambridgeshire). Future studies should seek to validate multiple questionnaires in the same sample in order to test which of the questionnaires is the most reliable, valid and also feasible. The current study explored interaction by demographic variables such as sex, ethnicity and language on the relationship between the two measures. These variables were not found to impact the association between the EY-PAQ and accelerometry within the present sample.

The error for both MVPA and ST were lowest when mean accelerometer MVPA and ST were highest. This meant that parents were more likely to over or under report MVPA and ST using the EY-PAQ when children’s accelerometer-determined levels of MVPA and ST were low. This finding could be explained by the different measurement properties of a proxy-report questionnaire and accelerometer. Dependent upon the placement of the monitor, accelerometry constantly measures movements of the child when worn. However, proxy-report questionnaires are reliant upon what parents observe and remember when completing the questionnaire. Parents will not remember every 15 s of movement or sedentary time that their young child has engaged in throughout the day, but the accelerometer (in the current study) recorded the child’s movement (or lack thereof) every 15 s (one of the epoch lengths used). Therefore, a difference between the two measurements was foreseeable. Despite the differences between the two types of methods, accelerometers are the most widely used comparison measure in which new self-report tools are validated against [30] Because of the differences, strong coefficients are seldom reported, with most self-reported validity coefficients with accelerometry ranging between 0.25 and 0.41 [30].

In the current study, the inclusion of boundaries informed by data from a recent systematic review [15] narrowed the limits of agreement and greater accuracy was observed. However, it has to be noted this could lead to a possible exclusion of participants, and reduced sample sizes if the EY-PAQ was applied to future population-level studies. In this study, the percentage of participant loss due to the application of boundaries was 23% (*n* = 47) for MVPA and 30% (*n* = 58) for ST. Nonetheless, large scale studies using accelerometry also need to factor in participant loss due to the processing of data (e.g., not enough valid wear time). In comparison to large scale studies using accelerometry, the EY-PAQ’s participant loss was similar to that of the Healthy Active Preschool Years study (30%) [46], and less than half of the Millennium Cohort Study (children aged 7–8 years) (53%) [47]. The use of the EY-PAQ and accelerometry share the limitation of possible sample size reduction, but the implementation of accelerometry and other objective measures are limited by the burden to participants (e.g., seven days wearing of monitor), level of expertise required to process data and financial costs, all which do not apply to the EY-PAQ. The results of the current study indicate that the EY-PAQ has the smallest limits of agreement after the application of boundaries when measuring MVPA compared to other similar questionnaires [27,44].

It seems that the EY-PAQ is not suitable for measuring young children’s ST. The Pre-PAQ also measures ST in young children, and like the EY-PAQ, it was also found to be a weak measure. Reasons for inaccuracy could include that accelerometry would have likely detected most sedentary behaviours including those not measured by the EY-PAQ (e.g., bathroom and meal times). However, the two techniques are also measuring two different behaviours. The EY-PAQ measures the time and frequency that parents report their child sitting down and doing different activities. Accelerometry measures ST by quantifying the absence of movement. Another reason for inaccuracy could be the design of the EY-PAQ’s questions. For example, parents rarely used free-text responses to report engagement in sedentary behaviours ≥60 min, but it is known that, even in this young age group, TV-viewing is prevalent and possibly prolonged. By having a response category with a maximum of 60 min daily, and free-text responses thereafter, we may have unintentionally given parents the impression that we would not have expect higher values, thereby dissuading truthful responses. Future research should bear this in mind and investigate validity and reliability parameters when using different and more suitable comparison measures (e.g., inclinometers [48]) and adding more domains of ST within the EY-PAQ (e.g., time spent sitting while eating).

A strength of the current study was the relatively large sample of children under the age of five years compared to previous validation studies in young children [27,44]. Furthermore, the EY-PAQ sample were from a low socio-economical, ethnically diverse and bi-lingual population; thereby, this studies’ novelty adds to the current measurement literature of young children’s PA and ST, which is heavily skewed by white English speaking samples [27]. However, results from the current study may not be generalisable to other populations; therefore, additional validity and reliability studies using this new measure in different populations are required. Although a widely used objective field measure, the use of accelerometers as a comparison measure for ST was a limitation; they do not detect posture and only estimate ST through a lack of movement counts. Accelerometry therefore could be argued to be an unsuitable convergent measure for ST [48]. However, there is currently no other objective measure of habitual ST that has been used with children of this age [49]. Finally, (in)activity levels show variation throughout the day in young children [50], meaning that missing data from particular segments of the day (morning, afternoon, or evening) may have influenced our objective data. In this respect, imparting a time-distribution caveat to the accelerometry data may have been worthwhile [51]. Nonetheless, ≥360 min of data has commonly been used to define a valid day of accelerometry in young children [14,32,39,52,53], without specifying the pattern of observed data, and our work has shown only small differences in estimated activity levels in the source population with and without specifying that all parts of days must be observed [54].

## 5. Conclusions

The EY-PAQ has acceptable reliability and validity for measuring habitual MVPA of young children from a bi-lingual (English, Urdu), bi-ethnic (White British, South Asian) low socio-economic community. In situations when objective methods are not possible for measurement of MVPA in young children, the EY-PAQ may be a suitable alternative, but only if boundaries are applied. Having such a questionnaire means researchers can explore the early life determinants of MVPA in an ethnically diverse and low socio economic status population at a low cost. Such evidence will be useful for the development of tailored interventions, with better chances to decrease health inequalities in PA and related health outcomes in young children.

## Figures and Tables

**Table 1:** Demographic characteristics of children in the validity and reliability analyses of the study.

Demographic and Outcome Variables	All *		Validity **				Reliability	
	*n* = 196		ST (*n* = 89) ***		MVPA (*n* = 102) ****		*n* = 104	
	*n* (%)	Mean (SD)	*n* (%)	Mean (SD)	*n* (%)	Mean (SD)	*n* (%)	Mean (SD)
**Sex**								
Boys	99 (50.5)		47 (52.8)		56 (54.9)		52 (48)	
Girls	97 (49.5)		42 (47.2)		46 (45.1)		57 (53)	
**Ethnicity**								
White British	82 (41.8)		30 (33.7)		37 (36.3)		33 (30)	
South Asian	96 (49)		52 (58.4)		55 (53.9)		67 (62)	
Other	18 (9.2)		7 (7.9)		10 (9.8)		9 (8)	
**Age**								
Years		3.2 (0.8)		3.2 (0.8)		3.2 (0.8)		3.3 (0.8)
**Language**								
English	156 (79.6)		69 (77.5)		84 (82.4)		82 (75)	
Urdu	40 (20.4)		20 (22.5)		18 (17.6)		27 (25)	
**EY-PAQ**								
Proportion estimates (%)				47.0 (13.6)		21.2 (11)		
**Actigraph GT3X+**								
Wear time (min per day)				594.8 (100.7)		582.1 (127.3)		
Minutes per day				344.1 (88.0)		118.4 (7.5)		
Proportion estimates (%)				57.5 (7.9)		20.3 (7.6)		

* Values presented are for validity analysis before the application of boundaries. ** Values presented are for the remaining sample after the application of boundaries. *** Costa cut-points (≤5 counts per 5 s). **** Pate cut-points (≥420 counts per 15 s).

**Table 2:** Intraclass-correlations for moderate to vigorous physical activity (MVPA) and sedentary time (ST) measured by the early years physical activity questionnaire (EY-PAQ).

EY-PAQ Test 1 *vs.* Test 2	Reliability			
	n(%)	Mean Daily Difference in Minutes [95% CI]	ICC (2,1)	95% Confidence Interval
MVPA	104 (93.7)	25.5 [−23.9, 74.8]	0.35 *	0.17–0.50
ST	104 (93.7)	1.7 [−36.5, 39.9]	0.47 *	0.3–0.61

* *p* ≤ 0.05.

**Table 3:** Validity of the early years physical activity questionnaire (EY-PAQ) compared to accelerometry.

EY-PAQ *vs.* Accelerometer	Validity						
	N (%)	rho	Mean Daily Difference in Minutes [95% CI]	LOA ^†^	Error Correlations (r)	Heteroskedasicity *p*-Value	Ratio LOA ^†^
MVPA: No boundaries	149 (76.0)	0.03	106.3 [72.5, 140.2]	−303.7 to 516.4	−0.80 *	0.94	1.20 (×/÷ 10.6)
MVPA: Boundary applied >2% (16.8 min)<41% (344.3 min)#	102 (52.0)	0.30 *	7.1 [−12.3, 26.4]	−185.9 to 200.1	−0.37 *	<0.01	1.03 (×/÷ 5.8)
ST: No boundaries	147 (75.0)	0.02	−160.0 [−189.1, −30.9]	−509.9 to 190.0	−0.67 *	<0.01	1.72 (×/÷ 3.6)
ST: Boundary applied >30% (252 min) <94% (789.6 min) #	89 (45.4)	0.19	−87.5 [−117.6, −57.4]	−367.6 to 192.7	−0.50 *	<0.01	1.26 (×/÷ 1.9)

* *p* ≤ 0.05. # Hnatiuk *et al.* [17] boundaries. ^†^ Limits of agreement.

## Supplementary Materials

The following are available online at http://www.mdpi.com/2075-4663/4/2/30/s1. Appendix A: Early Years Physical Activity Questionnaire English Version; Appendix B: Early Years Physical Activity Questionnaire Urdu Transliteration Version.

## Abbreviations

The following abbreviations are used in this manuscript:

EY-PAQ: Early Years Physical Activity Questionnaire
TPA: Total Physical Activity
MVPA: Moderate to Vigorous Physical Activity
ST: Sedentary Time
BiB: Born in Bradford
