# Supplementary material for: Reliability and Validity of the Early Years Physical Activity Questionnaire (EY-PAQ)
Source: Sports (Basel). 2016 May 26;4(2):30. doi: 10.3390/sports4020030 (PMC5968919; doi:10.3390/sports4020030)
Supplement: Supplementary file 1 [file sports-04-00030-s001.zip › Appendix B_Early Years Physical Activity Questionnaire Urdu Transliteration Version.pdf]

Study ID: ____________
APPENDIX B

Early Years Physical Activity Questionnaire

Hello mera naam ………………….hain, mai Born in Bradford se hoon. Aap ka bohut shukriya ke aap humare saat mill rahi hai. Hum yeh jaana chahte hai, ke sargarmiya karte huway, aur TV dekhte huway aap ka bacha/bachee ne kitna waqt guzzara/guzaari, pichle mahine ke ekh aam hafte mai. Hum aapke shukar guzaar honge agar aap sab sawaalon ke jawaab de sakay, albata agar aap kuch sawaalon ke jawaab nahi dena chahte toh yeh bhi theek hai. Koi sahi yah galat jawaab nahi hai. Agar koi bhi jawaab dene mai aapko pareshaani ho, toh aap humay sawaal poochne main hichkichaana nahi. Aap ke saare sawaal bharosemand honge. Aap ka naam aur pata iss sawaalnaame main kahin bhi nahi likha jayga. Hum maafi chahte hai, agar koi bhi sawaal aapko buray lagay.

Example: Pichle mahine main, kitne din aor kitni dair tak aap keh sakti ho ke aap ka bacha ne in jismani sargarmiyaan karta ho ga ghar main ? (Meharbani kar ke nishan lagain)

	Less than once a week	How often	For how long each day<br>Please tick one box or write a time if more than one hour daily			
	Yes/ No	Number of days each week. If never put a zero	Up to 15 mins/day	16-30 mins/day	31-60 mins/day	More than an hour/day. Please estimate time
Colouring/drawing/crafting	No	5/7				Hrs: 2 Mins: 30
Sitting playing with toys (e.g. dolls/puzzles/educational play)	No	7/7			✓	Hrs:….. Mins:…..
Watching TV/DVDs	No	7/7				Hrs: 1 Mins: 45
Playing on the computer (do not include physically active games such as Nintendo Wii)	Yes	…../7		✓		Hrs:….. Mins:…..

Aap ka buhot shukriya in sawaalon ka jawaab denay ka

Study ID: ____________
Questionnaire completed by (please circle)**:** Mother / Father / Other (please specify):

Q1. Pichle mahine main, kitne din aor kitni dair tak aap keh sakti ho ke aap ka bacha ne in jismani sargarmiyaan karta ho ga ghar main ? (Meharbani kar ke nishan lagain)

	Hafte mein eik se kam	Kitni baar	Har din Kitni dair tak<br>Please tick one box or write a time if more than one hour daily			
	Yes/ No	Hafte mai Kitni Din<br>If never put a zero	Din mai Pandra minute	Din mai Solan se Thees minute	Din mai Adhay ghante se ekh ghanta	Din mai ekh ghante se ziyada<br>(takriban kitna samay)
Tasweron main rang bharte/taswer banate		**…../7**				Hrs:….. Mins:…..
Beth kar khilonon se kheltay (e.g. dolls/puzzles educational play)		**…../7**				Hrs:….. Mins:…..
TV ya DVD dekhtay huway		**…../7**				Hrs:….. Mins:…..
Computer per kheltay huway (not physically active games such as Nintendo Wii)		**…../7**				Hrs:….. Mins:…..
Beth kar gana suntay/gana gatay howay		**…../7**				Hrs:….. Mins:…..
Parhtay howay ya kisi ko parhta suntay howay		**…../7**				Hrs:….. Mins:…..
Ghar main uchal kood karte howay kheltay howay (nachna, ghutnon per chalna, dorna, khilonon per bethna ya sawari karna, push toys, physically active computer games such as Nintendo Wii)		**…../7**				Hrs:….. Mins:…..

Study ID: ____________

Ghar ke sehan ya garden main kheltay huway		**…../7**				Hrs:….. Mins:…..
Ya aisi jismani sargarmiyan jinhain karne se us ki sans phol jati ho ya mehnat karni parti ho		**…../7**				Hrs:….. Mins:…..

Q2. 2. Pichle mahine, aik jagha se dosri jagha jatay howay (maslan dukanain, school ya groups, park, doston ya rishtedaron ke ghar), aap kya kaho gi kay aap kay bache ne aik haftay main kitne din aur ekh din main kitne dair ke liya guzaara:

	Hafte mein eik se kam	Kitni baar	Har din Kitni dair tak<br>Please tick one box or write a time if more than one hour daily			
	Yes/ No	Hafte mai Kitni Din<br>If never put a zero	Din mai Pandra minute		Yes/ No	Hafte mai Kitni Din<br>If never put a zero
buggy/pushchair main		**…../7**				Hrs:….. Mins:…..
Chaltay howay		**…../7**				Hrs:….. Mins:…..
Kisi ki goud main		**…../7**				Hrs:….. Mins:…..
Gahri main		**…../7**				Hrs:….. Mins:…..
public transport main		**…../7**				Hrs:….. Mins:…..

Study ID: ____________

Q3. In the last month, on how many days each week and for how long each day has your child done the following?

	Hafte mein eik se kam	Kitni baar	Har din Kitni dair tak<br>Please tick one box or write a time if more than one hour daily			
	Yes/ No	Hafte mai Kitni Din<br>If never put a zero	Din mai Pandra minute		Yes/ No	Hafte mai Kitni Din<br>If never put a zero
Played at the park/playground		**…../7**				Hrs:….. Mins:…..
Played at Indoor play facilities (e.g. ball pools)		**…../7**				Hrs:….. Mins:…..
