# Supplementary material for: Reliability and Validity of the Early Years Physical Activity Questionnaire (EY-PAQ)
Source: Sports (Basel). 2016 May 26;4(2):30. doi: 10.3390/sports4020030 (PMC5968919; doi:10.3390/sports4020030)
Supplement: Supplementary file 1 [file sports-04-00030-s001.zip › Appendix A_Early Years Physical Activity Questionnaire English Version.pdf]

Study ID: ____________

APPENDIX A

Loughborough University | BRADFORD INSTITUTE FOR HEALTH RESEARCH | BORNINBRADFORD

Early Years Physical Activity Questionnaire

Please complete this questionnaire when your child has worn their physical activity monitor for 7 days. The questionnaire asks about the types of activities your child has been doing in the last 4 weeks, how often and for how long. There are three parts. There are no right or wrong answers and all responses are confidential. Please answer all questions, unless there are questions that you do not wish to answer, you can skip these. If you are unsure about any of the questions please ask a member of school staff or the research team for guidance when you return the questionnaire and activity monitors.

Example answers… In the last month, how many days each week and for how long each day would you say your child has spent doing the following activities at home?

	Less than once a week	How often	For how long each day Please tick one box or write a time if more than one hour daily			
	Yes/ No	Number of days each week. If never put a zero	Up to 15 mins/day	16-30 mins/day	31-60 mins/day	More than an hour/day. Please estimate time
Colouring/drawing/crafting	No	5/7				Hrs: 2 Mins: 30
Sitting playing with toys (e.g. dolls/puzzles/educational play)	No	7/7			✓	Hrs:..... Mins:.....
Watching TV/DVDs	No	7/7				Hrs: 1 Mins: 45
Playing on the computer (do not include physically active games such as Nintendo Wii)	Yes	...../7		✓		Hrs:..... Mins:.....

Thank you in advance for completing the questionnaire

Questionnaire completed by (please circle)**:** Mother / Father / Other (please specify):

Study ID: ____________

<u>Q1. In the last month, how many days each week and for how long each day would you say your child has spent doing the following activities at home?</u>

	Less than once a week	How often	For how long each day<br>Please tick one box or write a time if more than one hour daily			
	Yes/ No	Number of days each week. If never put a zero	Up to 15 mins/day	16-30 mins/day	31-60 mins/day	More than an hour/day, please estimate time
Colouring/drawing/Craft		**…../7**				Hrs:….. Mins:…..
Sitting playing with toys (e.g. dolls/puzzles/educational play)		**…../7**				Hrs:….. Mins:…..
Watching TV/DVDs		**…../7**				Hrs:….. Mins:…..
Playing on the computer (do not include physically active games such as Nintendo Wii)		**…../7**				Hrs:….. Mins:…..
Sitting listening/singing to music		**…../7**				Hrs:….. Mins:…..
Reading/being read to		**…../7**				Hrs:….. Mins:…..
Playing actively inside the house (e.g. dancing, crawling, running, sit and ride toys, push toys, physically active computer games such as Nintendo Wii)		**…../7**				Hrs:….. Mins:…..
Playing actively in the garden/yard		**…../7**				Hrs:….. Mins:…..
Engaging in physical activity/active play that makes them sweat or breathe harder		**…../7**				Hrs:….. Mins:…..

Study ID: ____________

<u>Q2. In the last month, to get from place to place (e.g to the shops, school/groups, park, visiting friends/relatives), on how many days each week and for how long each day would you say your child has spent:</u>

	Less than once a week	How often	For how long each day<br>Please tick one box or write a time if more than one hour daily			
	Yes/ No	Number of days <u>each</u> week. If never put a zero	Up to 15 mins/day	16-30 mins/day	31-60 mins/day	More than an hour/day, please estimate time
In their buggy/pushchair		**…../7**				Hrs:….. Mins:…..
Walking		**…../7**				Hrs:….. Mins:…..
Being carried		**…../7**				Hrs:….. Mins:…..
In the car		**…../7**				Hrs:….. Mins:…..
On public transport		**…../7**				Hrs:….. Mins:…..

<u>Q3. In the last month, on how many days each week and for how long each day has your child done the following?</u>

	Less than once a week	How often	For how long each day<br>Please tick one box or write a time if more than one hour daily			
	Yes/ No	Number of days <u>each</u> week. If never put a zero	Up to 15 mins/day	16-30 mins/day	31-60 mins/day	More than an hour/day, please estimate time
Played at the park/playground		**…../7**				Hrs:….. Mins:…..
Played at Indoor play facilities (e.g. ball pools)		**…../7**				Hrs:….. Mins:…..
